# The development and validation of a scale to explore staff experience of governance of economic efficiency and quality (GOV-EQ) of health care

**DOI:** 10.1186/s12913-018-3765-7

**Published:** 2018-12-12

**Authors:** Sara Korlén, Anne Richter, Isis Amer-Wåhlin, Peter Lindgren, Ulrica von Thiele Schwarz

**Affiliations:** 1 Medical Management Centre, LIME, Karolinska Institutet, 171 77 Stockholm, Sweden; 2 The Swedish Institute for Health Economics, Box 2017, 220 02 Lund, Sweden; 3 School of Health, Care and Social Welfare, Mälardalen University, Box 883, 721 23 Västerås, Sweden

**Keywords:** Scale development, Factor analysis, Health care governance, Economic incentives, Quality, Health care staff, Motivation

## Abstract

**Background:**

In publicly funded health care systems, governance models are developed to push public service providers to use tax payers’ money more efficiently and maintain a high quality of service. Although this implies change in staff behaviors, evaluation studies commonly focus on organizational outputs. Unintended consequences for staff have been observed in case studies, but theoretical and methodological development is necessary to enable studies of staff experience in larger populations across various settings. The aim of the study is to develop a self-assessment scale of staff experience of the governance of economic efficiency and quality of health care and to assess its psychometric properties.

**Methods:**

Factors relevant to staff members’ experience of economic efficiency and quality requirements of health care were identified in the literature and through interviews with practitioners, and then compared to a theoretical model of behavior change. Relevant experiences were developed into sub-factors and items. The scale was tested in collaboration with the Department of Rehabilitation Medicine at a university hospital. 93 staff members participated. The scale’s psychometric properties were assessed using exploratory factor analysis, analysis of internal consistency and criterion-related validity.

**Results:**

The analysis revealed an eight factor structure (including sub-factors *knowledge and awareness*, *opportunity to influence*, *motivation, impact on professional autonomy* and *organizational alignment*), and items showed strong factor loadings and high internal consistency within sub-factors. Sub-factors were interrelated and contributed to the prediction of impact on clinical behavior (criterion).

**Conclusions:**

The scale clearly distinguishes between various experiences regarding economic efficiency and quality requirements among health care staff, and shows satisfactory psychometric quality. The scale has broad applications for research and practice, as it serves as a tool for capturing staff members’ perspectives when evaluating and improving health care governance. The scale could also be useful for understanding the underlying processes of changes in provider performance and for adapting management strategies to engage staff in driving change that contributes to increased economic efficiency and quality, for the benefit of health care systems, patients and staff.

**Electronic supplementary material:**

The online version of this article (10.1186/s12913-018-3765-7) contains supplementary material, which is available to authorized users.

## Background

Health care systems share the challenge of increasing demands and limited resources [1]. In publicly funded systems, governing bodies struggle to design governance models that push public service providers to attain economic efficiency and quality, to optimize the value of tax payers’ money. This has resulted in increased provider competition and the introduction of financial incentive models, particularly in primary care [2]. Hospitals, commonly under public ownership, are under a general pressure to cut costs by reducing annual budgets [3].

Concurrent with the management of financial constraints, health care systems need to meet quality targets. Although formal definitions of quality in health care include efficient resource use, the common understanding of the concept of quality centers around improving the population’s health outcomes and its experience of care [4]. Specific (soft) governance initiatives encourage health care providers to monitor and improve quality, through e.g. national quality registers, quality assessments and benchmarking of providers [5]. In addition, financial incentive models often include quality targets to ensure that health care providers meet quality standards [6, 7]. Perspectives on quality in health care governance are important for (at least) two reasons. First, it is an important target outcome on its own right, as part of the triple aim of health care [4]. Second, it must be ensured that efforts to increase economic efficiency do not have unintended consequences for quality. Therefore, the economic efficiency and quality requirements are highly interrelated in health care provision, and economic governance models can’t be evaluated, or understood, without perspectives on quality (and vice versa).

The application of economic governance models relies on the assumption that ultimately, they will change the performance of provider organizations. Nevertheless, although this assumption implies changes in staff behavior, evaluation studies in the field of governance and policy commonly focus on organizational outputs, such as cost, resource use, productivity and (more rarely) patient outcomes [8–10]. Therefore, existing studies do not capture the underlying causes of change in organizational outputs, such as changes in staff behavior, nor do they uncover the processes involved. Behavioral change theories (e.g., COM-B) [11] can help in understanding the effectiveness by which governance models influence staff behavior. The COM-B model synthesizes several theories of behavior change and identifies interrelated variables central in initiating and maintaining new behaviors over time. The model postulates that behavior change is a function of individuals’ knowledge and skills (*competence*); the processes, resources and the social environment provided by the organization (*opportunity*); and the motivational drivers that initiate and maintain the desired behaviors over time (*motivation*). Thus, the COM-B model can be used to structure influential factors according to their relevance to behavior change and could be useful to understand behavior change in relation to health care organizations’ external demands.

To our knowledge, the application of theoretical models to understand how economic governance models influence staff behavior is rare. Some empirical studies focus on staff members’ experiences of specific governance and policy applications, commonly taking a qualitative case study approach and highlighting consequences for care delivery and the risk of unintended negative consequences for patients and staff [12–14]. These studies show the relevance of exploring staff perspectives when investigating the implications of governance models, yet there is a lack of methods and studies capturing the experience of larger and more representative populations, which would enable comparisons across groups and over time. For this purpose, self-assessment scales are considered a valid method to capture subjective experiences in larger populations [15]. The literature on economic incentives presents survey studies using self-assessment scales at the staff level [16, 17]. However, these scales focus on experiences with specific incentive models, which make items less relevant when transferred to other health care settings. To sum, self-assessment scales that capture a more general experience of governance are needed for use in larger populations. Furthermore, there is a need for scales that capture a comprehensive perspective on governance, integrating the demands of economic efficiency and quality, and include experiences of theoretical relevance for behavior change.

## Aim

The aim of this study is to develop and assess the psychometric properties of a self-assessment scale measuring staff members’ experience of economic efficiency (subscale A) and quality requirements (subscale B) of health care.

Acknowledging governance as a complex concept, it has been defined as the actions taken by governing bodies to influence providers of public services in specific directions [18, 19]. Within the scope of this study, governance is defined as the general requirements of economic efficiency and quality of health care provider services. In addition, we focus on how these requirements are experienced at the staff level, including individuals’ perceptions, thoughts and feelings. In this study, the concept of economic efficiency is distinguished from quality, and when addressing quality we refer to the common understanding of quality focusing on desirable patient outcomes and experiences of care.

## Method

The current study consists of two phases, beginning with the scale development phase aiming at identifying appropriate staff experiences to include in the scale. Throughout the development phase we applied a broad perspective on governance and made efforts to include sources of knowledge representing a variety of health care settings and professional perspectives. Thereafter, the scale’s psychometric properties were assessed by pilot testing the scale in a public university hospital setting.

### Scale development

**Fig. 1:**
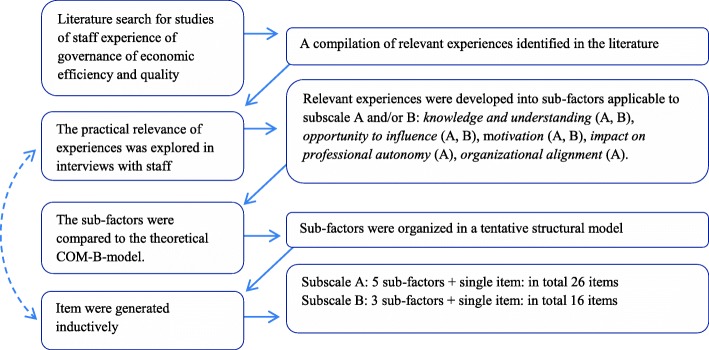
An illustrative overview of the developmental process

The scale development process followed general recommendations described by Hinkin [20] and is illustrated in Fig. 1.

#### Literature search

First, we searched the databases Web of Science and Scopus for studies including self-assessment scales capturing staff experience of governance models to increase economic efficiency and quality. Examples of experiences found in the literature are shown in Table 1. Key terms such as “self-assessment scale”, “scale”, “self-assessment measure”, “measure” and “survey” were used in combination with key terms such as “economic incentives”, “incentive-model”, “reimbursement model”, “economic governance” (for subscale A) and key terms such as “quality evaluation”, “quality improvement” and “quality governance” (for subscale B). Additional searches were also made in Google scholar, in relevant grey literature and by manually studying reference lists of identified articles.

**Table 1:** Sub-factors applied to Subscales A & B and examples from the literature and interviews

Sub-factors for Subscale A and B	Examples of related experiences in the literature	Quotes from the exploratory interviews
• Knowledge and understanding (A & B): The knowledge and understanding of how economic efficiency/quality requirements affect how his/her work is to be conducted.	*Awareness and understanding* [17] *Information and analysis* [22] *Planning for quality* [23]	A: “If I don’t meet enough patients we get less money. So that’s very obvious. I think everyone knows and has an awareness of being a part of it.” *(Respondent 5)* B: “Quality follow-up. That’s done a lot right now. There are different ways. At the work place meetings. Some have white boards where they take notes continuously. And print graphs to share. They are educational and easy for staff to follow.” *(Respondent 2)*
• Opportunity to influence (A & B): The experience of participating and being able to influence the financial situation/ quality improvement work at the unit.	*Control* [17] *Strategic planning for quality analysis* [22]	A: “They (staff) are not involved. You are not involved in the decision-making. You never get to know what financial resources there are, and what the costs are.” *(Respondent 2)* B: “So it is quite common that you measure quality in different ways, on the other hand there might be a lack of feedback. / ... / And if you get feedback it might not be obvious, what did I do that made a difference? Or you get feedback at a higher level of abstraction, and it’s difficult to know if you make a difference or not. You need to experience that you can influence quality.” *(Respondent 3)*
• Motivation (A & B): The experience of motivation and engagement in improving the unit’s financial situation/quality.	*Goal importance* [16] *Job satisfaction* [21] *Work load* [21] *Financial reward* [14] *Staff motivation* [13] *Human resource utilization analysis* [22] *Managerial role (Berlowitz* et al.*, 2003*	A: “My experience is also that, usually, healthcare workers do not really think about this (economic efficiency), or are engaged in this.” *(Respondent 7)* B: “Taking a quality perspective, I think it’s much easier to discuss, and it feels better than when you refer to money. Because people are not like that. Physicians, and all healthcare professionals, are guided by their ethics. It’s so central, you want to provide good care, and you’re in this to create good things for patients. To work for better health, that’s the intrinsic driving force. ”*(Respondent 1)*
• Impact on professional autonomy (A): The experience of economic efficiency requirements affecting his/her professional autonomy in meeting patient needs.	*Clinical relevance* [16] *Impact on professional autonomy* [16] *Impact on clinical behavior, clinical relevance* [17] *Impact on clinical roles, alignment professional values* [14] *Impact on clinical autonomy, changed clinical practice* [12]	A: “I feel that it is part of care, to do good /…/ It’s a driving force that’s part of who we are. It is in ethical perspectives we end up many times, in the ethical dilemmas. When I can’t do what I know is best for the patient. Although I would like to, and could, if the conditions were right.” *(Respondent 3)*
• Organizational alignment (A): The experience that the unit’s financial resources are in line with its mission.	*Impact on quality of care* [21] *Unintended consequences for patients* [17] *Supporting improvement, unintended consequences for patients* [14] *Fairness, appropriateness* [13]	A: “It is always so, that we (health care providers) should do more using fewer resources. And it seems to me that the more promises of that kind that you throw into the political game, the better. But then what? /…/ Then we have to provide the same services, with less money.” *(Respondent 4)*

Regarding economic governance, we identified two short self-assessment scales [16, 21] and one extensive scale [17] capturing experiences of financial incentive models in professional organizations. As the available scales were few, additional searches for case studies were conducted using the key words above (for subscale A) in combination with “case-study” and “qualitative study”. Several studies exploring staff members’ experiences of specific incentive models were identified [12–14, 21]. We could not identify any scales that specifically addressed staff members’ experiences of the governance of quality. However, we identified scales capturing staff experience of work on quality improvement [22, 23] that included relevant aspects of quality evaluation. By reviewing and synthesizing previous studies’ findings we identified commonly observed experiences that could constitute the scales’ sub-factors.

#### Qualitative interviews to explore sub-factors

To explore the appropriateness of using the sub-factors in the self-assessment scale, we conducted semi-structured interviews (*n* = 7) with health care staff members, with experience from a variety of health care settings. To cover multiple professional perspectives respondents were recruited through the national associations for physicians, nurses and midwifes. One nurse, one midwife and two physicians volunteered. To add a provider perspective we recruited one manager and one nurse from a local primary care facility. Last, we interviewed two civil servants at the county council level who were experienced in issues of health care governance.

The first author conducted the interviews, which lasted 45–60 min, from April to June 2017. The interviews were conducted face-to-face (*n* = 5) or on the phone (*n* = 2). All but two respondents (county council representatives) were interviewed individually. All interviews were recorded on a digital tape-recorder and transcribed. The interviews were analyzed deductively, applying a thematic approach [24] and using the software Nvivo. Predefined codes were constructed in correspondence with sub-factors, to which relevant data were linked. Additional codes were created for relevant data that did not match the predefined codes. The sub-factors were reviewed in relation to interview data to identify experiences (sub-factors) that were present in the daily practice and that respondents could easily relate to. The first author conducted the analysis and discussed it with the remaining authors. Guided by the analysis, we derived three sub-factors from the literature that were relevant and applicable for both subscales: *knowledge and understanding*, *opportunity to influence* and *motivation*. Two additional sub-factors, primarily derived from studies of economic efficiency, were found relevant for subscale A: *impact on professional autonomy* and *organizational alignment*. The selected sub-factors are presented and defined in Table 1, including illustrative quotes from the exploratory interviews.

#### Applying a theoretical model

**Fig. 2:**
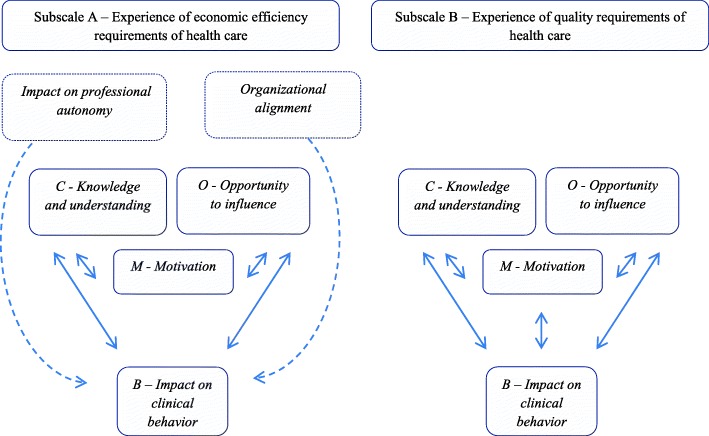
A tentative structural model of sub-scales and sub-factors

The COM-B model was used to assess the theoretical relevance of sub-factors for behavior change. The sub-factors were mapped to the COM-B components and all were found to be theoretically relevant. Three sub-factors corresponded well to COM-B components (*knowledge and understanding*, *opportunity to influence* and *motivation).* The sub-factors *impact on professional autonomy* and *organizational alignment* were, to our judgement, related to multiple components of the model and therefore remained unclassified. A tentative structural model of sub-factors for sub-scale A and B was created, illustrated in Fig. 2.

#### Item generation

An inductive item generation approach was applied, which is recommended when available scales are scarce [25]. A summative rating scale format was chosen [26], in which items are formulated as statements that respondents judge based on agreement, using the commonly applied 5-point Likert scale format [26]. The first author generated an extensive item pool, inspired by available scales and interview data. The first author then developed items iteratively in collaboration with the remaining authors and discussed them with research colleagues. Because at least three items are recommended for a reliable and valid sub-scale [26], the purpose was to generate four to six items for each sub-factor, to enable exclusion of low-quality items. Aware of the debated pros and cons of reversed items [15], we created at least one reversed item per sub-factor. We also created a single item for both subscales to measure *impact on clinical behavior* in relation to economic efficiency and quality requirements, to be used as criterion variables in the assessment of criterion-related validity.

We pilot-tested the finalized items in collaboration with one physician and two psychologists. They received the survey by email and provided written feedback by email (*n* = 1) or in face-to-face meetings (*n* = 2). The feedback resulted in minor revisions, and all authors collaborated to finalize items. At this stage of development the total scale (single items excluded) consisted of 40 items, including 25 items in subscale A and 15 items in subscale B. Last, we included background questions and an open ended question for additional comments at the end of the survey [27].

### Pilot testing and validating the GOV-EQ-scale

#### Study setting

We pilot tested the scale in collaboration with the Department of Rehabilitation Medicine (RM) at a public university hospital in Sweden. The Department of RM is organized into four clinical units, which provide inpatient- and outpatient treatment and rehabilitation for patients suffering from medical conditions such as stroke, spinal injury and brain injury. The staff is organized in multi-disciplinary teams and includes a broad range of professional expertise, such as occupational therapists, speech therapists, psychologists, physiotherapists, physicians, social workers, nurses and assistant nurses.

The hospital is reimbursed through annual budgets and the hospital management team distributes resources to departments considering input from local management. No financial incentives are linked to specific activities or outcomes, however, the economic situation is strained and all departments are generally required to reduce costs. The quality of services is evaluated by continuous quality evaluation by the local provider organization, national benchmarking in quality registers, and external evaluation of compliance with international standards for best practices and quality in medical rehabilitation (CARF).

#### Data collection

Before sending out the survey, we conducted cognitive interviews with volunteering members of staff (*n* = 2) and unit managers (n = 2) at the RM units, to collect verbal information about the response process [28]. We applied a retrospective approach, in which participants filled out the survey independently and then described their understanding of the items [29]. We also formulated anticipated probes, i.e. questions, about words that we found particularly challenging [30]. For practical reasons the first author sent the survey by email and followed up with phone interviews. We summarized individual talk aloud reports for each respondent. The feedback resulted in minor revisions of three items, consisting of change of word order and replacing one word with a more neutral one. The respondents supported the clustering of items from the same sub-factor to increase comprehension [31]. We made minor revisions to the instructions to increase clarity.

We distributed the final survey on paper to all staff members (*n* = 183), excluding those on parental- and sick leave. The survey included a letter from the researchers informing the participants about the project’s aim and that participation was anonymous and could be withdrawn at any time. It further clarified that the return of a completed survey implied informed consent to participate in this research study. Staff members received additional information about the study through the hospital’s intranet and in their weekly team meetings. Completed surveys were returned in sealed, anonymous envelopes to the unit managers and stored temporarily until the first author collected them. The data collection period started in November 2017 and was closed after 6 weeks. Two reminders were sent out by email to all staff members and the unit managers gave oral reminders in staff meetings.

#### Sample characteristics

Of 183 persons receiving the survey 93 participated. This implies a response rate of 51%, which is in line with survey studies in health care settings [32]. Missing data on item level was low (< 2.2%). Data from two individuals were deleted based on a large proportion of missing data (59 and 86%), resulting in a final sample of 91 participants. A summary of the study population’s characteristics is presented in Table 2.

**Table 2:** Sample characteristics (*n* = 91)

Demographic characteristics		n=	%
Gender	Women	76	84
	Men	13	14
	Other	1	1
	Missing	1	1
Professional role	Occupational therapist	10	11
	Social worker	6	7
	Speech therapist	3	3
	Physician	4	4
	Psychologist	8	9
	Nurse	8	9
	Assistant nurse	22	24
	Physiotherapists and other	29	32
	Missing	1	1
Experience as health care manager	Yes	12	13
	No	76	84
	Missing	3	3
		Mean	SD
Age	Years	43.7	12.4
Experience from working in health care	Years	16.7	12.0

#### Statistical analysis

Two people independently digitalized survey data, and controlled for discrepancies (< 3%) in relation to original data. Data was imported into SPSS 25, which was used for all statistical analyses. The psychometric properties of the scales were tested using exploratory factor analysis (EFA), reliability analysis and assessment of criterion validity following general guidelines of scale development [15, 20] and specific guidelines for exploratory factor analysis [33–35]. An EFA was chosen because we primarily derived sub-factors from empirical studies [33]. Based on our ambition to identify factors of theoretical relevance, we used a common factor model (principal axis factoring, PAF) [34]. Oblique factor rotation (direct oblim) was used, as we expected factors to correlate [33]. Data was checked for suitability by inspecting inter-item correlations, using *r* > .3 as a criteria for inclusion, and using Bartlett’s Test of Sphericity (BTS) and the Kaiser-Meyer-Olkin measure of sampling adequacy (KMO) [36].

The Kaiser criteria of Eigenvalues > 1 and visual inspection of the scree plot were used to determine the appropriate number of factors [20]. We assessed the relevance of retained factors by inspecting communalities (preferably > 0.6) [37] and total variance explained, considering 60% as a minimum acceptable target [20]. Last, we applied our qualitative judgement to evaluate factors’ appropriateness in relation to the intended factor structure [34].

For items, we inspected the pattern matrix and included items with factor loadings > .3 on to a single factor not suffering from cross-loadings on to other factors (> .3) [36]. Cross-loading items were accepted if loadings were at least > .4 and twice as strong in the assigned factor, as in other factors. The internal consistency (i.e. reliability) of items assigned to a factor was assessed using Cronbach’s alpha, considering .7 as a minimum acceptable level [20]. The inter-factor relationships were explored by correlating all factors’ mean-based indices. To assess criterion-related validity a multiple regression model (Ordinary Least Squares Regression) was calculated for subscales A and B separately, using the sub-factors in each subscale as predictor variables and the single items measuring *impact on clinical behavior* as dependent (criterion) variable (item 11 and item 37 respectively).

#### Translation

All items have been translated from Swedish to English for the purpose of this paper, applying a multi-step approach recommended in the literature [38, 39]. First, a professional translator conducted a forward translation. Thereafter, two researchers, bilingual in Swedish and English and knowledgeable of the health care context, met with the first author to assess the conceptual equivalence of translated and original items. Minor revisions to the translated items were decided upon in consensus. Last, a professional language editor checked the final version.

## Results

### Exploring dimensionality and items

Two items did not fulfil the criteria of inter-item correlations >.3 and were dropped prior to factor analysis. The data’s general suitability was supported by a KMO index >.5 (.67) and a significant BTS (*p* < .001). The factor analysis (PAF, direct oblim rotation) initially resulted in a nine factor solution. Eight factors corresponded well with the intended factor structure, but the ninth factor included a number of reversed items not loading onto factors as expected. Based on our qualitative judgement the ninth factor was found inconsistent and did not make a meaningful contribution to the scale, which is why we dropped three items loading strongly in Factor 9 that did not make a substantial contribution to other factors. The factor analysis was re-run and an eight factor solution was identified (presented in detail in Additional file 1), based on the criteria of eigenvalues > 1 and a visual inspection of the scree plot. The eight factors accounted for 71.8% of variance explained and communalities were strong (>.5) for a majority (78%) of items.

All items had factor loadings >.3 and all items were assigned to a factor. The vast majority of items did not suffer from problematic cross-loads (>.3). However, items 14, 16 and 21 (presented in Table 3) showed negative cross-loads across factors 2 and 7, indicating that those factors were related. Based on our qualitative judgment of item content, the less serious nature of cross-loads and the expected association of sub-factors, we decided to keep these items and assign them to the factor in which they showed stronger factor loadings and were found most relevant. Two items [6, 32] did not load on the expected factor but were found to make an appropriate contribution to the designated factor based on item content.

**Table 3:** Items and sub-factors of the final scale including mean, SD, Cronbach’s alpha values and factor loadings

Sub-factors and items (of subscale A/B)	Cronbach’s Alpha	Mean (SD)	F1	F2	F3	F4	F5	F6	F7	F8
Knowledge and awareness (A)	0.86	2.99 (.91)								
1. I know how I should take the unit’s financial situation into consideration in my work.		3.26 (1.19)						−.802		
2. I know what I can do to make the unit’s financial situation as good as possible.		3.09 (1.17)						−.809		
3. I know how to deal responsibly with the unit’s financial resources.		3.26 (1.18)						−.711		
4. I know how to plan my work to ensure that we stay within the unit’s budget.		2.58 (1.21)						−.786		
5. I find it difficult to see how I can influence the unit’s financial situation (R).		2.54 (1.30)						−.500		
6. I am aware of the unit’s financial situation when I make decisions in my work with patients.		3.22 (1.06)						−.326		
Opportunity to influence (A)	0.89	1.97 (.95)								
7. I get involved in discussions concerning the unit’s financial situation.		2.07 (1.12)				−.795				
8. I can influence how the financial resources are used in the unit.		1.77 (1.07)				−.925				
9. I am able to express my opinions on how we can use the unit’s resources more efficiently.		2.37 (1.09)				−.728				
10. My opinions matter when budgetary decisions are made.		1.72 (1.07)				−.838				
Motivation (A)	0.83	3.08 (1.01)								
11. It’s motivating to work with issues that concern the unit’s financial situation.		2.54 (1.19)					.822			
12. It’s fulfilling to try to improve the unit’s financial situation.		3.51 (1.18)					.803			
13. I am interested in the unit’s financial situation.		3.22 (1.16)					.693			
Impact on professional autonomy (A)	0.62	3.31 (.92)								
14. The unit’s financial status affects my ability to do what is best for patients.		3.53 (1.19)							.499	
15. The unit’s financial limitations affect my ability to adhere to my own ethical values.		2.97 (1.26)							.638	
16. I feel free to do what is best for the patient, regardless of the unit’s financial situation (R).		3.44 (1.21)							.315	
Organizational alignment (A)	0.87	2.29 (.84)								
17. I think the unit’s financial resources are reasonable.		2.27 (.99)		.830						
18. We have the financial resources needed to meet patient needs.		2.26 (1.07)		.831						
19. I think the unit’s financial situation is sustainable.		2.19 (.91)		.906						
20. The unit’s financial status is sufficient to allow us to fulfill our mission.		2.30 (1.01)		.739						
21. The financial requirements placed on the unit negatively impact our patients. (R)		2.36 (1.16)		.568						
Single item: Impact on clinical behavior (A)										
22. I take the unit’s financial situation into consideration in my clinical work.		3.32 (1.00)	.342				.389			
Knowledge and awareness (B)	0.82	4.06 (.72)								
23. I know what leads to good quality care for our patients.		4.09 (.88)	0.637							
24. I know what I should do, in my role, to ensure that we maintain high levels of quality.		4.35 (.72)	0.821							
25. I know how I can get involved in quality improvement.		3.59 (1.14)	0.454							
26. I know how to plan my work to ensure that what I do is of good quality.		4.21 (.80)	0.877							
Opportunity to influence (B)	0.89	3.31 (.91)								
27. I can influence how the unit works with quality improvement.		3.10 (1.17)			−.817					
28. I participate in the unit’s work with quality improvement.		3.40 (1.18)			−.687					−.340
29. I can influence where we focus our improvement work.		3.01 (1.16)			−.933					
30. My opinions matter when we work with quality improvement.		3.13 (1.09)			−.834					
31. By the time we begin our work on quality improvement, it has already been decided how it should be carried out. (R)		3.28 (1.08)			−.622					
32. I find it difficult to see how I can influence quality at the unit. (R)		3.85 (1.07)			−.377					
Motivation (B)	0.78	4.15 (.69)								
33. Quality improvement work is motivating.		4.11 (.94)								−.762
34. I think it is part of my role to get involved with quality improvement.		4.27 (.73)								−.763
35. It’s fulfilling to try to improve quality at the unit.		4.32 (.92)								−.310
36. I am interested in how we compare to other units with regard to quality.		3.89 (.96)								−.338
Single item: Impact on clinical behavior (B)										
37. I take quality into consideration in my clinical work.		4.24 (.85)	.561							

Notes: Factor loadings (PAF, direct oblim) for items on to assigned factors. Factor loadings <.3 are omitted from the Table. (R) indicates that the item is reversely scored

All sub-factors showed satisfactory levels of internal consistency, with the exception of *impact on professional autonomy,* which did not meet the Cronbach’s alpha criteria. With regard to this being a newly developed scale, and to enable further improvement, we decided to keep this sub-factor and items. The single item of subscale A showed cross-loadings (>.3) in two factors, indicating its independence from other factors. For subscale B, the single item loaded only on one factor, but based on the expected relationship between factors and behavior change we decided to keep the single item for the assessment of criterion-related validity. The final version of the scale contains two subscales (A and B), including five sub-factors with a total of 21 items (subscale A) and three sub-factors with a total of 14 items (subscale B). Items and sub-factors are presented in Table 3, including mean, SD, Cronbach’s alpha values and factor loadings in assigned factors. Single items are also included in the table. Factor loadings <.3 are omitted.

### Inter-factor relationships

Table 4 presents means and correlations for all sub-factor indices. As expected based on theory, several sub-factors were moderately related to each other. The interrelationships between *knowledge and awareness, opportunity to influence* and *motivation* were stronger for subscale B, than for subscale A. The sub-factors *impact on professional autonomy* and o*rganizational alignment* in subscale A were not related to any other sub-factors but were negatively correlated to each other. The sub-factors *knowledge and awareness* and *motivation* were moderately correlated across subscales A and B, however not for the sub-factor *opportunity to influence*.

**Table 4:** Mean, SD and inter-factor correlations of sub-factor indices

Sub-scale	Index	M (SD)	1.	2.	3.	4.	5.	6.	7.	8.
A	1. Knowledge and awareness	2.99 (.91)	1							
A	2. Opportunity to influence	1.97 (.95)	.398^**^	1						
A	3. Motivation	3.08 (1.01)	.220^*^	.215^*^	1					
A	4. Impact on professional autonomy	3.31 (.92)	−.065	−.167	−.096	1				
A	5. Organizational alignment	2.29 (.84)	.055	.187	.019	−.475^**^	1			
B	6. Knowledge and awareness	4.06 (.72)	.452^**^	.152	.081	−.091	−.152	1		
B	7. Opportunity to influence	3.31 (.91)	.094	.214^*^	.278^**^	−.256^*^	.114	.278^**^	1	
B	8. Motivation	4.15 (.69)	.129	.051	.415^**^	.070	−.174	.450^**^	.474^**^	1

Notes: Pearson correlation, *p < .05** *p* < .01 (2-tailed)

### Criterion-related validity

For subscale A, the multiple regression model (df (5, 79) = F 9.24, *p* < .001) showed that sub-factors *knowledge and awareness* and *motivation* contributed significantly to predict the dependent (criterion) variable *impact on clinical behavior,* explaining 37% of the variance. Similarly, for subscale B the multiple regression model (df (3, 84) = F 18.47, p < .001) showed that sub-factors *knowledge and awareness* and *motivation* made significant contributions to predict *impact on clinical behavior*, with an explained variance of 40%. Remaining sub-factors made no significant contribution. Results from the multiple regression analysis for subscales A and B are presented in Additional files 2 and 3.

## Discussion

The aim of the study was to develop a scale that measures staff members’ experience of governance of economic efficiency and quality of health care and assess its psychometric properties. The analyses show that the s*taff experience of governance of economic efficiency and quality (GOV-EQ) scale* distinguishes between eight interrelated experiences and holds good psychometric qualities. The GOV-EQ scale can contribute to the understanding of how governance of health care is perceived among staff and can help determine the likeliness of staff behavior change in accordance with the demands at the provider level.

The eight-factor structure identified in the factor analysis shows that the GOV-EQ scale can capture various experiences of economic efficiency and quality requirements, which implies that staff members have opinions and can assess these issues. Although studies of staff experience are rare in the empirical literature on economic governance of health care, our findings support the feasibility of conducting such studies. According to our results, staff members seem moderately knowledgeable of economic efficiency requirements, and motivated to engage in improving the unit’s financial situation, which implies that engaging staff in driving change to find better use of resources is possible. However, staff members experience few opportunities to influence issues related to economic efficiency, which (at least in theory) impair the conditions for driving change [11]. In addition, empirical studies confirm the positive effects of increasing staff involvement in budgetary issues to establish a more positive attitude among professionals to consider economic efficiency in clinical work [40] and to increase financial performance at the unit level [41]*.* In this study, the low levels of influence can be interpreted as staff members considering issues of economic efficiency out of their control or even the unit’s control. However, this could also reflect a need among staff members to be more involved and have a closer dialogue with management about these issues.

In line with previous literature [12, 14], our findings show that economic efficiency requirements to some extent limit the experience of professional autonomy among staff. Those who consider themselves restricted in meeting patient needs also experience lower degrees of alignment between financial resources and the organization’s mission. Although this relationship could be expected, none of these sub-factors are associated with the overall motivation to engage in improving the financial situation at the unit level. Therefore, the experience of imbalance between resources and patient needs does not necessarily make staff less engaged in improving economic efficiency of care delivery. This finding suggests a complex interplay of experiences at staff level, which merits further exploration. Still, the measures of staff experience of organizational alignment and professional autonomy present opportunities to monitor risks of unintended consequences of economic efficiency requirements, which has been proposed as an important component of improving health care governance [2]. To sum, our results support the relevance of exploring staff perspectives on economic governance further, to better understand the complexity of staff experience. A growing body of empirical data will gradually increase our understanding of how assessments of staff experiences should be interpreted and understood.

The levels of knowledge, motivation and opportunity to influence are generally higher regarding quality requirements, compared to economic efficiency. In theory, this means that the conditions for involving staff in driving change to improve quality are better than for engaging them in improving economic efficiency. Because previous literature proposes that satisfying patient needs [42] and improving care [43] are key components of health care staff motivation, this might not come as a surprise. However, regarding economic efficiency, staff experiences relatively few opportunities to influence quality issues, which can be interpreted as the involvement of staff members being a general challenge for management.

The experiences of being knowledgeable and motivated to improve quality are associated with their counterparts regarding economic efficiency requirements. Therefore, individuals that are more aware of how to improve quality also are more aware of how to increase services’ economic efficiency. Although correlations leave no room for conclusions about causal effects, these results suggests that taking an integrated approach to economic efficiency and quality requirements could have synergetic effects. If efforts were made to engage staff members in quality improvement, the motivation to reduce waste and use resources more efficiently could come as a natural part of improvement work. In addition, if units face requirements of increased economic efficiency, the integration of perspectives on quality could diminish the risk of reducing resource use at the expense of patients. To sum, our findings show that staff members’ experiences of governance regarding economic efficiency and quality are interrelated. Consequently, the GOV-EQ scale reveals opportunities to gain a broader understanding of the overall implications of governance. Still, specific sub-factors (and items) could be selected for particular purposes.

### Methodological considerations

The GOV-EQ scale relies on robust methodology, integrating empirical findings with perspectives from theory and practice. We believe this combination contributes to the scale’s relevance and usefulness, which has broad applications in future studies that could contribute to the understanding of the implications of governance in health care. A valid self-assessment scale enables studies of larger populations, in which experiences of various forms of governance can be studied and compared over time. In evaluation studies, data on staff members experience could be related to other measures of organizational outputs, to unveil the processes explaining why provider performance improves (or degrades) as a result of governance reforms.

Although the GOV-EQ scale shows promising psychometric properties, the scale’s overall validity needs to be interpreted in the light of potential methodological weaknesses. Based on general guidelines, which describe sample sizes of 100 as poor, 200 as fair and > 300 as good [35] this study’s sample size could be questioned. However, such guidelines are debated [33, 34] since studies have revealed that strong data, displaying a clear factor structure and strong factor loadings, can make a smaller sample size adequate [34]. Communalities also play a critical role and levels > 0.5–0.6 can make factor-analytic solutions reliable also for samples well below 100 [44]. To sum, although the sample size can be considered disadvantageous, the clear factor structure, overall strong factor loadings and high communalities provide arguments for relying on the factor analysis results. Still, this methodological weakness needs to be acknowledged and the need for improvements should be addressed by developing weak items further, in particular for less robust sub-factors (*impact on professional autonomy* and *motivation* related to quality). In addition, a very important next step is to confirm the scale’s factor structure in a Confirmatory Factor Analysis based on supplementary data.

In the scale development process we made efforts to increase the content validity of the scale by interviewing representatives from the target group at several steps of the development process. For the scope of this study we conceptualize governance as health care staff members’ experiences of economic efficiency and quality requirements. Although we have made extensive efforts to explore staffs members’ understanding of the scale and make adjustments, there is a risk that these concepts may be too abstract and hard to judge for individual staff members. Another risk arises from the fact that understanding varies depending on the model of governance and the provider context. To ensure items’ content validity in future studies we recommend having a close dialogue with the provider organizations and staff members prior to using the scale, and if necessary, make adjustments to increase staffs members’ understanding of the items. Important next steps are to further translate and validate the scale in other languages.

Regarding the case’s representativeness and the results’ generalizability, the study setting key characteristics include the handling of complex care delivery in a public hospital setting and staff members working in multidisciplinary teams. The provider organization is under general economic pressure, but without specific incentives linked to targets or activities. The study population consists of various professional groups and several clinical units. Even though it may be assumed the scale’s psychometric properties are valid in similar health care settings, the generalizability of the study results should be established in future studies in additional provider settings. Additionally, the use of the GOV-EQ scale should include an a priori analysis of its suitability based on how governance models are manifested in daily practice and how staff members experience economic efficiency- and quality requirements in clinical work.

### Implications for research

The findings in this study have several implications for future research in the field. From a methodological perspective, additional studies are needed to explore the GOV-EQ scale’s reliability and validity in other settings and in other languages. Opportunities exist to improve the scale’s psychometric properties. To increase the scale’s time efficiency, a shorter version of the scale could be developed and validated, including only three items per sub-factor.

We believe the scale’s relevance for behavior change could be increased by expanding the application of theory in future studies. The assessment of criterion validity showed that only a number of sub-factors significantly contributed to the prediction of behavior change. Although our approach presents limitations, in particularly regarding the single items’ validity, we believe that the scale’s relevance for behavior change could be improved by expanding the use of theory in an additional development step. Additional sub-factors could be developed deductively from theoretical models, which could make the scale more comprehensive from a behavioral perspective. For example, the COM-B components, which are multi-faceted in nature, could be reviewed for additional themes that could be developed into sub-factors (and items), incorporating factors ranging from individual capabilities to organizational resources and social environments. Also, additional theoretical models of behavior could be explored for suitability.

This study presents opportunities for empirical studies in the field of governance research and we believe that the subjective nature of staff members’ experience should be further explored. What individual and contextual factors that shape staff members’ experiences of reforms at the systems level are most likely as much a result of how such models are communicated and implemented, as of the formal model requirements [45]. The role of interaction between management and staff, and staff members’ beliefs and assumptions about the agendas of management and policy makers are interesting topics to explore further. Given the matter’s complex nature, we further encourage mixed method approaches [46] when researchers use the GOV-EQ scale, to enrich the interpretation of results by collecting additional qualitative data.

### Implications for practice

The GOV-EQ scale could also be useful to practitioners, by increasing the understanding of governance implications at the macro, meso and micro level, not only to improve specific governance models but also to inform the local adaption and implementation of governance requirements in provider organizations. The scale could be used to determine whether staff members consider specific targets defined at the provider- or department level feasible, and whether targets are communicated in an understandable and engaging manner. For individual managers, information about staff members’ experiences could be monitored and used to fine-tune their own leadership strategies to enhance staff knowledge, involvement and engagement in driving change.

## Conclusions

This study provides a self-assessment scale that measures staff members’ experiences of governance in regard to economic efficiency and quality requirements. The scale’s psychometric properties are satisfactory, but they must be confirmed in studies in additional health care settings. The scale can contribute to the understanding of governance implications and increase the understanding of how local management in provider organizations can transform external requirements of economic efficiency and quality into feasible tasks and targets that involve and engage staff members in driving change to improve health care delivery.

## Additional files

Additional file 1: **Appendix A:** Final factor solution, including eigenvalues and variance explained. (DOCX 15 kb)

Additional file 2: **Appendix B:** Multiple regression analysis of sub-factors predicting impact on clinical behavior (Subscale A). (DOCX 14 kb)

Additional file 3: **Appendix C:** Multiple regression analysis of sub-factors predicting impact on clinical behavior (Subscale B). (DOCX 14 kb)

## Abbreviations

EFA: Exploratory factor analysis
PAF: Principal Axis Factoring
RM: Rehabilitation Medicine
