# Supplementary material for: The development and validation of a scale to explore staff experience of governance of economic efficiency and quality (GOV-EQ) of health care
Source: BMC Health Serv Res. 2018 Dec 12;18:963. doi: 10.1186/s12913-018-3765-7 (PMC6292102; doi:10.1186/s12913-018-3765-7)
Supplement: Supplementary file 1 — Appendix A: Final factor solution, including eigenvalues and variance explained. (DOCX 15 kb) [file 12913_2018_3765_MOESM1_ESM.docx]

**Appendix A:** Final factor solution, including eigenvalues and variance explained.

| **Number, name of sub-factor and sub-scale** | **Eigenvalues** | **% of variance** | **Cumulative %** |
| --- | --- | --- | --- |
| F1 Knowledge and understanding, B | 8.1 | 21.7 | 21.7 |
| F2 Organizational alignment, A | 5.2 | 14.2 | 36.0 |
| F3 Opportunity to influence, B | 4.2 | 11.4 | 47.4 |
| F4 Opportunity to influence, A | 2.6 | 7.0 | 54.4 |
| F5 Motivation, A | 2.1 | 5.6 | 60.0 |
| F6 Knowledge and understanding, A | 1.7 | 4.6 | 64.6 |
| F7 Impact on professional autonomy, A | 1.6 | 4.3 | 68.9 |
| F8 Motivation, B | 1.1 | 3.0 | 71.9 |
