# Supplementary material for: The development and validation of a scale to explore staff experience of governance of economic efficiency and quality (GOV-EQ) of health care
Source: BMC Health Serv Res. 2018 Dec 12;18:963. doi: 10.1186/s12913-018-3765-7 (PMC6292102; doi:10.1186/s12913-018-3765-7)
Supplement: Supplementary file 2 — Appendix B: Multiple regression analysis of sub-factors predicting impact on clinical behavior (Subscale A). (DOCX 14 kb) [file 12913_2018_3765_MOESM2_ESM.docx]

**Appendix B:** Multiple regression analysis of sub-factors predicting impact on clinical behavior (Subscale A).

| Predictor | Coefficients | 95 % CI [LL, UL] |
| --- | --- | --- |
| *Knowledge and awareness* | .394** | [.22, .66] |
| *Opportunity to influence* | .118 | [-.09, .34] |
| *Motivation* | .310** | [.12, .49] |
| *Impact on professional autonomy* | .127 | [-.09, .37] |
| *Organizational alignment* | .051 | [-.18, .30] |
| *R^2^* | .369** |  |

Notes: ** p< .01; all coefficients are standardized.
